# Molecular mechanisms of sex determination in Lepidoptera: current status and perspectives

**DOI:** 10.1111/1744-7917.70111

**Published:** 2025-07-02

**Authors:** František Marec, Atsuo Yoshido, Arjen E. van′t Hof

**Affiliations:** ^1^ Biology Centre of the Czech Academy of Sciences Institute of Entomology České Budějovice Czech Republic

**Keywords:** convergent evolution, dosage compensation, feminizing piRNA, *Masculinizer*, moths and butterflies, sex‐determining pathway

## Abstract

Moths and butterflies (Lepidoptera) are the largest group of organisms with female heterogamety and the sex chromosome system WZ/ZZ (female/male) or exceptionally Z0/ZZ. However, the genetic basis of sex determination in Lepidoptera remained unknown for a long time until the sex‐determining pathway was discovered in 2014 in the silkworm *Bombyx mori*. In this species, the dominant W chromosome carries a *Feminizer* (*Fem*) gene encoding a precursor of a *Fem* piRNA that promotes femaleness by downregulating the expression of a Z‐linked gene, *Masculinizer* (*Masc*). In the W chromosome absence, *Masc* promotes male development and controls dosage compensation. In the 10 years since this discovery, significant progress has been made in understanding the molecular mechanisms of sex determination in Lepidoptera. Data from recent studies discussed in this review suggest a conserved role for *Masc* in male sex determination and dosage compensation in the clade Ditrysia, which comprises the majority of Lepidoptera. Although the primary sex‐determining signals are not conserved, the presence of feminizing piRNAs of different origins in distantly related species suggests convergent evolution of a similar mechanism of female sex determination. A unique exception is zygosity‐based sex determination in the butterfly *Bicyclus anynana*, where the primary signal is the state of the hypervariable *Masc* gene. In other species with a dispensable W chromosome, such as the silkmoth *Samia cynthia*, sex is determined by the Z:A ratio, but a molecular mechanism is not yet known. Overall, the available data suggest considerable diversity in the upstream molecular mechanisms of sex determination in Lepidoptera.

## Graphical abstract

The genetic basis of sex determination in Lepidoptera was discovered in 2014 in the silkworm *Bombyx mori*. In this model species, the W chromosome‐derived small piRNA called *Fem* piRNA downregulates the expression of a Z‐linked gene, *Masculinizer* (*Masc*), which leads to the default female‐specific splicing of the *doublesex* gene (*dsx^F^*) and thus to female development. In the W chromosome absence, the Masc protein promotes male‐specific splicing of the *doublesex* gene (*dsx^M^*), leading to male development. Masc also controls dosage compensation. Recent data from several species suggest that *Masc* plays a conserved role in male sex determination and dosage compensation in most Lepidoptera, whereas the primary sex‐determining signals are not conserved and remain unknown in most species studied. However, the discovery of different feminizing piRNAs in distantly related species suggests convergent evolution of a similar sex‐determining pathway. A special case is the sex determination in the butterfly *Bicyclus anynana*, in which WZ individuals with one *Masc* allele develop into females and ZZ individuals with two different *Masc* alleles develop into males, but ZZ homozygotes for *Masc* die as embryos. In the silkmoth *Samia cynthia* with a dispensable W chromosome, sex is determined by the Z:A ratio, but a molecular mechanism is not yet known. The available data suggest a conserved function of the *Masc* and *doublesex* genes but considerable diversity in the primary signals of sex determination in Lepidoptera.

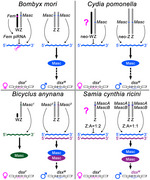

## Introduction

Most insect groups have sex chromosome systems with male heterogamety, XY/XX or X0/XX (male/female) (Traut, 1999; Blackmon *et al.*, 2017). However, moths and butterflies (Lepidoptera) have a system with female heterogamety, referred to as WZ/ZZ or Z0/ZZ (female/male), depending on whether the W chromosome is present or not (Traut *et al.*, 2007). The Z0/ZZ system is thought to be an ancestral system, as it occurs in the sister order Trichoptera (caddisflies) and in basal groups of Lepidoptera (Traut & Marec, 1997; Marec & Novák, 1998; Dalíková *et al.*, 2017; Hejníčková *et al.*, 2019). With the exception of the basal groups (see fig. 5. in Hejníčková *et al.*, 2019), most of the moths and butterflies studied so far have a WZ/ZZ system of chromosomal sex determination (Traut *et al.*, 2007; Sahara *et al.*, 2012; Zrzavá *et al.*, 2018). Variations of this system include species with multiple W or Z chromosomes or with both, as well as sporadic cases of species that have lost the W chromosome. The multiple sex chromosomes, such as WZ_1_Z_2_ or W_1_W_2_Z, usually result from the fusion of an autosome with the ancestral Z or the ancestral W chromosome, forming a neo‐Z (Z_1_) or a neo‐W (W_1_) sex chromosome, while the free autosome becomes the Z_2_ or W_2_ sex chromosome. In addition, sex chromosome‐autosome translocations and fusions may also contribute to the evolution of more complex systems of multiple sex chromosomes in Lepidoptera (Marec *et al.*, 2010; Yoshido *et al.*, 2013; Šíchová *et al.*, 2015; Šíchová *et al.*, 2016; Yoshido *et al.*, 2020; Hejníčková *et al.*, 2021). Some species, such as the monarch butterfly *Danaus plexippus*, or even entire groups of Lepidoptera, such as the species‐rich superfamilies Tortricoidea and Gelechioidea, have an apparently standard WZ/ZZ system, which, however, consists of neo‐sex chromosomes formed by fusion of the ancestral sex chromosomes with a pair of autosomes (Yoshido *et al.*, 2011; Nguyen *et al.*, 2013; Šíchová *et al.*, 2013; Mongue *et al.*, 2017; Picq *et al.*, 2018; Carabajal Paladino *et al.*, 2019; Rueda‐M *et al.*, 2024).

Consistent with female heterogamety, two main mechanisms of chromosomal sex determination have been proposed for Lepidoptera: (1) the dominant W mechanism with a primary signal gene (i.e., a female‐determining gene) on the W chromosome and (2) the Z‐counting mechanism with a male‐promoting gene on the Z chromosome (Traut *et al.*, 2007; Sahara *et al.*, 2012). In the first case, sexual development depends only on the presence or absence of the W chromosome, whereas in the second case, sex is controlled either by the dose of Z chromosomes alone or by the balance between Z chromosomes and autosomes, that is, the Z:A ratio.

In insects, sex determination is controlled by a gene cascade that was originally discovered in *Drosophila melanogaster*. In this cascade, each gene controls the expression or splicing of the next gene from top to bottom. In *Drosophila*, the cascade includes the *Sex‐lethal* (*Sxl*), *transformer* (*tra*), *transformer‐2* (*tra‐2*), *doublesex* (*dsx*) and *fruitless* (*fru*) genes, in addition to the primary signal, which is either the X chromosome‐to‐autosome ratio (X:A) or, according to an alternative model, the number of X chromosomes (reviewed in Saccone, 2022). However, this cascade is only partially conserved in other insects, namely Diptera, Hymenoptera and Coleoptera (Bopp *et al.*, 2014; Geuverink & Beukeboom, 2014). In particular, the primary signal genes at the top of the sex determination cascade undergo rapid evolutionary change (Saccone, 2022).

Since the beginnings of genetic research, the most important model for the study of sex determination in Lepidoptera has been the silkworm *Bombyx mori*, a fully domesticated insect species used industrially for silk production (Tazima, 1964). The history of studies on sex determination in *B. mori* was recently summarized in Katsuma *et al.* (2018), so we will only briefly present the main findings here. In the silkworm, the sex chromosome system with heterogametic females (WZ) and homogametic males (ZZ) was described more than 100 years ago (Tanaka, 1916). Early experiments with polyploids induced by a heat shock during early embryogenesis then showed that *B. mori* has the dominant W mechanism of sex determination, that is, the presence of the feminizing factor (*Fem*) on the W chromosome determines female development, regardless of the number of Z chromosomes (Hashimoto, 1933). After that, there was no progress for many years until the sex determination gene cascade was discovered in *Drosophila* (Cline & Meyer, 1996). Several studies have shown that homologs of the *Drosophila* genes *Sxl*, *tra2*, *dsx*, and *fru* are found in *B. mori*, while the *tra* gene is not present in its genome. However, it was found that only the homolog of *dsx* (*Bmdsx*), the bottom gene of the sex‐determining cascade, plays a role in sex determination, while the other homologs do not (Ohbayashi *et al.*, 2002; Niimi *et al.*, 2006; Fujii & Shimada, 2007). The *Bmdsx* gene is sex‐specifically spliced and regulates sexual differentiation (Ohbayashi *et al.*, 2001; Suzuki *et al.*, 2001; Suzuki *et al.*, 2003) as in *Drosophila* and many other insects, including all Lepidoptera species studied (Geuverink & Beukeboom, 2014; Saccone, 2022). The function of *Bmfru* in male sexual behavior is conserved in *B. mori* as in *Drosophila*, but unlike *Drosophila fru*, *Bmfru* does not appear to play a crucial role in sex determination of the male brain, but rather acts as a regulator of sex‐differential expression of olfactory receptors in the antennae, suggesting partial functional divergence (Xu *et al.*, 2020; Ueno *et al.*, 2023). The other two genes have acquired various sex‐related functions, such as *Bm‐Sxl* in apyrene spermatogenesis (Sakai *et al.*, 2019) and *Bmtra‐2* in testis development (Suzuki *et al.*, 2012). Conversely, two other genes were identified encoding nuclear factors, *Bombyx* homolog of P‐element somatic inhibitor (BmPSI) and *Bombyx* homolog of IGF‐II mRNA binding protein (BmIMP), which specifically binds to *Bmdsx* and are involved in sex‐specific splicing of *Bmdsx* (Suzuki *et al.*, 2008; Suzuki *et al.*, 2010). All these findings indicated that the sex‐determining pathway in the silkworm is significantly different from *Drosophila*. However, the long search for a protein‐coding gene on the W chromosome that acts as the primary trigger for female development did not lead to the discovery of the molecular mechanism of sex determination in the silkworm (Suzuki, 2010). Instead, it was later found that a noncoding gene determines the sex of the silkworm (Kiuchi *et al.*, 2014).

## *Fem* piRNA/Masc sex‐determining pathway discovered in *Bombyx mori*

The W chromosome of *B. mori* lacks protein‐coding genes and contains predominantly retrotransposons and other repetitive elements (Abe *et al.*, 2005). This repetitive nature made the assembly of the W chromosome sequence very difficult until recently (Lee *et al.*, 2024). However, the W chromosome was found to be a source of female‐enriched PIWI‐interacting RNAs (piRNAs), 23–30 nucleotide long small RNAs that can silence transposon activity in the gonads of animals by interacting with PIWI proteins (Kawaoka *et al.*, 2011). This finding was the basis for the surprising discovery of the primary trigger of sex determination in the silkworm by Kiuchi *et al.* (2014). Kiuchi *et al.* (2014) performed in‐depth sequencing of the RNA transcripts expressed in female and male *B. mori* embryos and identified a 767‐base‐long transcript that was expressed only in female embryos. They found that this transcript is a precursor of a 29‐nucleotide‐long, female‐specific piRNA that is produced in large quantities. Through a series of experiments, the authors showed that this piRNA is required for female sex determination. They concluded that the W‐linked gene encoding the precursor transcript is the *Feminizer* (*Fem*) and that the *Fem* piRNA produced is the long‐sought primary trigger of female development in the silkworm.

In the search for the target of *Fem* piRNA, Kiuchi *et al.* (2014) identified a genomic locus with significant complementarity to the *Fem* piRNA sequence. This locus was part of exon 9 of a protein‐coding gene located on the Z chromosome. They named this new gene *Masculinizer* (*Masc*). The authors experimentally confirmed the cleavage of *Masc* mRNA at the predicted site in early embryos and demonstrated conclusively through further experiments that the *Masc* gene promotes male development. Consistent with this function, *Masc* exhibits sex‐biased expression during early embryogenesis. Before the onset of sex determination, *Masc* expression is the same in both sexes, then increases rapidly in male embryos and, after reaching a peak at the time of sex determination, gradually decreases to a level similar to that of female embryos. In contrast, the expression of *Masc* in female embryos gradually declines and remains at a low level (Kiuchi *et al.*, 2014).

**Fig. 1:**
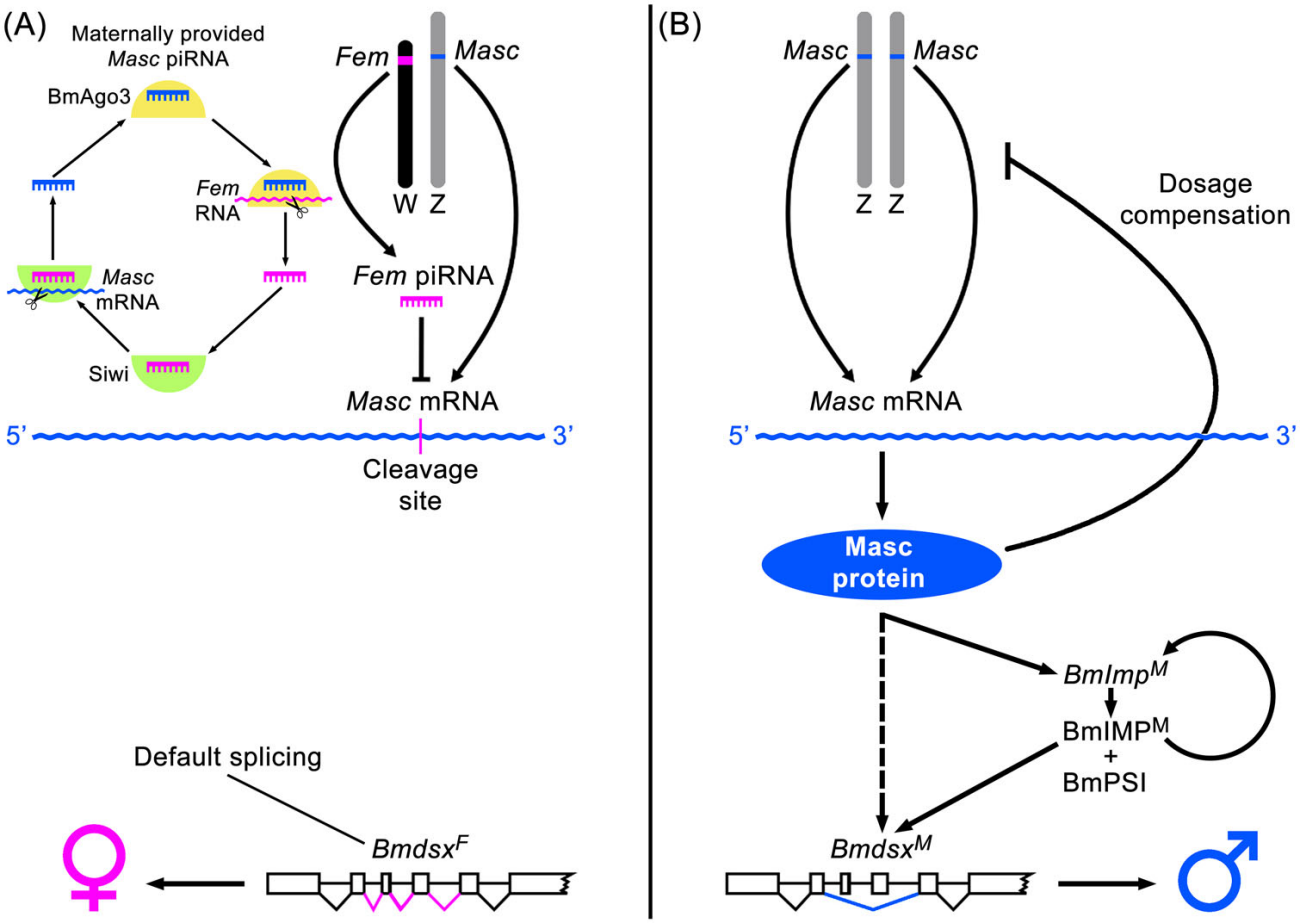
Sex‐determining pathway in *Bombyx mori*. (A) In WZ embryos, *Fem* piRNA, which is encoded by the W‐linked *Fem* gene, downregulates the expression of the Z‐linked *Masc* gene by cleavage of *Masc* mRNA. In the absence of the Masc protein, the default splice variant of the *Bmdsx* gene, *Bmdsx^F^*, then promotes female development. *Fem* piRNA is processed and amplified by the ping‐pong cycle, which is shown in simplified form in the upper left part of the panel (see text for details). (B) In ZZ embryos, *Masc* expression in the absence of the W chromosome leads to higher levels of the Masc protein, which induces the male splicing mode of the *BmImp* gene, *BmImp^M^*, resulting in the production of the BmIMP^M^ protein, which (i) maintains expression through an autoregulatory loop and (ii) forms a complex with the BmPSI protein. This complex alters the splicing of *Bmdsx* into the male‐specific form *Bmdsx^M^*, which promotes male development.

To summarize, the *Fem* piRNA/*Masc* pathway of sex determination in the silkworm discovered by Kiuchi *et al.* (2014) works according to the following scheme (Fig. 1). In WZ embryos, the *Fem* piRNA derived from the precursor RNA encoded by the W‐linked *Fem* cleaves *Masc* mRNA, preventing its translation into protein. In the absence of the Masc protein, the *Bmdsx* mRNA is then spliced by default in a female‐specific manner (*Bmdsx^F^*), resulting in female development (Fig. 1A). In ZZ embryos, translation of *Masc* mRNA into the Masc protein promotes male‐specific splicing of *Bmdsx*, leading to male development (Fig. 1B). In addition to its role in male sex determination, the Masc protein also induces dosage compensation through transcriptional downregulation of Z‐linked genes in ZZ individuals, that is, males (Tomihara *et al.*, 2022), which is achieved by partial repression of both Z chromosomes (Rosin *et al.*, 2022), similar to the dosage compensation of X‐linked genes in XX hermaphrodites of the nematode *Caenorhabditis elegans* (Ercan & Lieb, 2009). The exact mechanism by which Masc alters splicing of *Bmdsx* is not fully understood. The process is likely mediated by the male‐specific isoform of the *BmImp* gene, *BmImp^M^*, and the non‐sex‐specific expression of the *BmPsi* gene (Fig. 1B; Suzuki *et al.*, 2010; Sakai *et al.*, 2015). A higher level of Masc protein in ZZ embryos boosts the expression of *BmImp*, leading to its male‐specific splicing and the production of BmIMP^M^ protein. Once the BmIMP^M^ protein is produced, *BmImp^M^* expression is maintained by an autoregulatory function (Sakai *et al.*, 2015), which appears to be a common mechanism for regulating sex‐determining genes in insects, such as the *Sxl* gene in *Drosophila* females and *transformer* genes in females of some Diptera, Coleoptera and Hymenoptera (Sawanth *et al.*, 2016). The BmIMP^M^ and BmPSI proteins form a complex that binds to a cis‐regulatory element CE1 located in the female‐specific exon of *Bmdsx* and inhibits female‐specific splicing of *Bmdsx*, thereby promoting splicing to the male‐specific mode, *Bmdsx^M^* (Suzuki *et al.*, 2010). However, the role of *BmImp* was questioned by Xu *et al.* (2017), who found that CRISPR/Cas9‐mediated knockout of this gene did not affect sexual differentiation. The silkworm sex determination cascade likely relies on more than the five genes *Fem*, *Masc*, *BmImp*, *BmPsi*, and *Bmdsx*, as several other genes have been proposed to be potentially involved in the regulation of male‐specific splicing of *Bmdsx*, such as *Bmznf‐2*, which encodes a CCCH zinc finger protein, and *BxRBP3*, which encodes an RNA‐binding protein (Gopinath *et al.*, 2016; Zheng *et al.*, 2019; Yang *et al.*, 2021b; reviewed in Yang *et al.*, 2021a).

The Masc protein of *B. mori* has several characteristic domains (Katsuma *et al.*, 2015; Sugano *et al.*, 2016). Two CCCH‐type zinc finger domains at the N‐terminus were found to be dispensable for masculinization and dosage compensation and their role is currently unknown (Kiuchi *et al.*, 2019). A bipartite nuclear localization signal (bNLS) domain is essential for the nuclear localization of Masc, which is not associated with masculinization but may be crucial for dosage compensation, which is thought to occur in the nucleus (Sugano *et al.*, 2016). An 11 amino acid long masculinizing domain contains two conserved cysteine residues, Cys301 and Cys304, which are essential for masculinization (Katsuma *et al.*, 2015).

The *Fem* piRNA of *B. mori* is processed and amplified through a biogenesis pathway known as the ping‐pong cycle (Czech & Hannon, 2016). The entire process in *B. mori* has already been described in detail by Katsuma *et al.* (2014) and involves two PIWI proteins, *B. mori* Argonaute3 (BmAgo3) and silkworm Piwi (Siwi). Briefly, feminization is initiated by *Masc*‐derived piRNA, which is maternally provided to offspring (Kiuchi *et al.*, 2014). This *Masc* piRNA forms a complex with the BmAgo3 protein, which cleaves the *Fem* precursor RNA. The cleaved *Fem* RNA is bound by the Siwi protein and processed into a *Fem* piRNA‐Siwi complex, which cleaves the *Masc* mRNA, leading to the accumulation of *Masc* piRNA, which in turn forms a *Masc* piRNA‐BmAgo3 complex and continues the ping‐pong amplification loop (Fig. 1A). It should be emphasized that the amplification process relies on a 10‐nucleotide segment of sequence identity between *Fem* piRNA and *Masc* piRNA, referred to as the ping‐pong signature (Katsuma *et al.*, 2014; Kiuchi *et al.*, 2014). It should also be noted that the *Fem* gene is present in a high copy number on the W chromosome, depending on the silkworm strain (Katsuma *et al.*, 2018). A recent genome assembly of a *B. mori* female identified 129 *Fem* copies, arranged in tandem in one large and 10 smaller clusters on the W chromosome (Lee *et al.*, 2024).

## Conserved role of *Masc* in masculinization and dosage compensation

The discovery by Kiuchi *et al.* (2014) in the silkworm is the first example of a sex‐determining pathway controlled by the presence or absence of a piRNA. This discovery paved the way for research into the molecular mechanisms of sex determination in other lepidopteran species. One of the fundamental questions for subsequent studies was whether and to what extent the *Fem* piRNA/Masc pathway of sex determination is conserved in this large and diverse group of insects.

As expected, most studies in nonmodel species have focused on the protein‐coding *Masc* gene, which can be identified by homology‐based searches. Apart from the silkworm, the essential role of *Masc* in male development has so far been demonstrated in 11 other species from the same, distant and basal families (Table 1). The masculinizing function of the *Masc* gene thus appears to be conserved at least in the clade Ditrysia, which comprises about 98% of moths and butterflies (Kristensen & Skalski, 1999).

**Table 1:** Overview of lepidopteran species with experimentally proven function of the *Masculinizer* (*Masc*) gene in male sex determination

Superfamily Family	Species	Gene	Evidence	References
Bombycoidea				
Bombycidae	*Bombyx mori*	*Masc*	RNAi^†^	Kiuchi *et al.* (2014)
	*Trilocha varians*	*TvMasc*	RNAi^†^	Lee *et al.* (2015)
Noctuoidea				
Erebidae	*Hyphantria cunea*	*HcMasc*	CRISPR/Cas9^‡^	Li *et al.* (2024)
	*Lymantria dispar*	*LdMasc*	RNAi^†^	Moronuki *et al.* (2025)
Noctuidae	*Agrotis ipsilon*	*AiMasc*	CRISPR/Cas9^‡^	Wang *et al.* (2019b)
	*Helicoverpa armigera*	*HaMasc*	RNAi^†^	Deng *et al.* (2021)
Pyraloidea				
Crambidae	*Ostrinia furnacalis*	*OfMasc*	Wolbachia,^§^ CRISPR/Cas9^‡^	Fukui *et al.* (2015), Katsuma *et al.* (2022)
	*Ostrinia scapulalis*	*OsMasc^M^*, O*sMasc^F^*	Wolbachia^§^	Herran *et al.* (2023)
Pyralidae	*Ephestia kuehniella*	*EkMasc*, *EkMascB*	RNAi^†^	Visser *et al.* (2021)
Papilionoidea				
Nymphalidae	*Bicyclus anynana*	*BaMasc*	RNAi^†^	Van′t Hof *et al.* (2024)
Tortricoidea				
Tortricidae	*Cydia pomonella*	*CpMasc*	RNAi^†^	Pospíšilová *et al.* (2023)
	*Homona magnanima*	*HmMasc*	*Wolbachia* ^§^	Arai *et al.* (2025)
Yponomeutoidea				
Plutellidae	*Plutella xylostella*	*PxyMasc*	RNAi^†^	Harvey‐Samuel *et al.* (2020)

^†^ Knockdown by RNA interference (RNAi).

^‡^ Knockout by clustered regularly interspaced short palindromic repeats/CRISPR‐associated protein 9 (CRISPR/Cas9).

^§^ Downregulation by *Wolbachia* infection.

Another role of the *Masc* gene, namely dosage compensation of Z‐linked genes induced by the Masc protein, as shown in the silkworm (Katsuma *et al.*, 2019; Tomihara *et al.*, 2022), is closely related to its role in male sex determination. Therefore, the male‐specific embryonic lethality in the silkworm observed after depletion of *Masc* mRNA is most likely due to a failure of dosage compensation (Kiuchi *et al.*, 2014; Katsuma *et al.*, 2018). Masc‐induced dosage compensation has also been suggested for the closely related *Trilocha varians* (Katsuma *et al.*, 2019) and the distant *Plutella xylostella* and *Cydia pomonella* (Harvey‐Samuel *et al.*, 2020; Pospíšilová *et al.*, 2023). In addition, the induction of dosage compensation was experimentally confirmed for the *Ostrinia furnacalis* Masc protein (OfMasc). In *O. furnacalis*, the male‐killing factor Oscar, produced by the bacterial symbiont *Wolbachia*, binds to OfMasc and impairs its functions, that is, it inhibits masculinization and disrupts dosage compensation, resulting in male‐specific lethality (Fukui *et al.*, 2015; Katsuma *et al.*, 2022). Results from cell‐based assays in the related species *Ostrinia scapulalis* and in the distant tortricid moth *Homona magnanima* suggest that *Wolbachia*‐induced male killing by Oscar‐induced suppression of Masc, leading to feminization and disruption of dosage compensation, may be a common mechanism in Lepidoptera (Herran *et al.*, 2023; Arai *et al.*, 2024; Arai *et al.*, 2025). Recently, the feminization and lethality of *Bicyclus anynana* embryos homozygous for *BaMasc* alleles was also attributed to dysregulated dosage compensation (Van′t Hof *et al.*, 2024). Taken together, the available data strongly suggest that both functions of the Masc protein, that is, control of masculinization and dosage compensation, are conserved in evolutionarily distant Lepidoptera species.

**Fig. 2:**
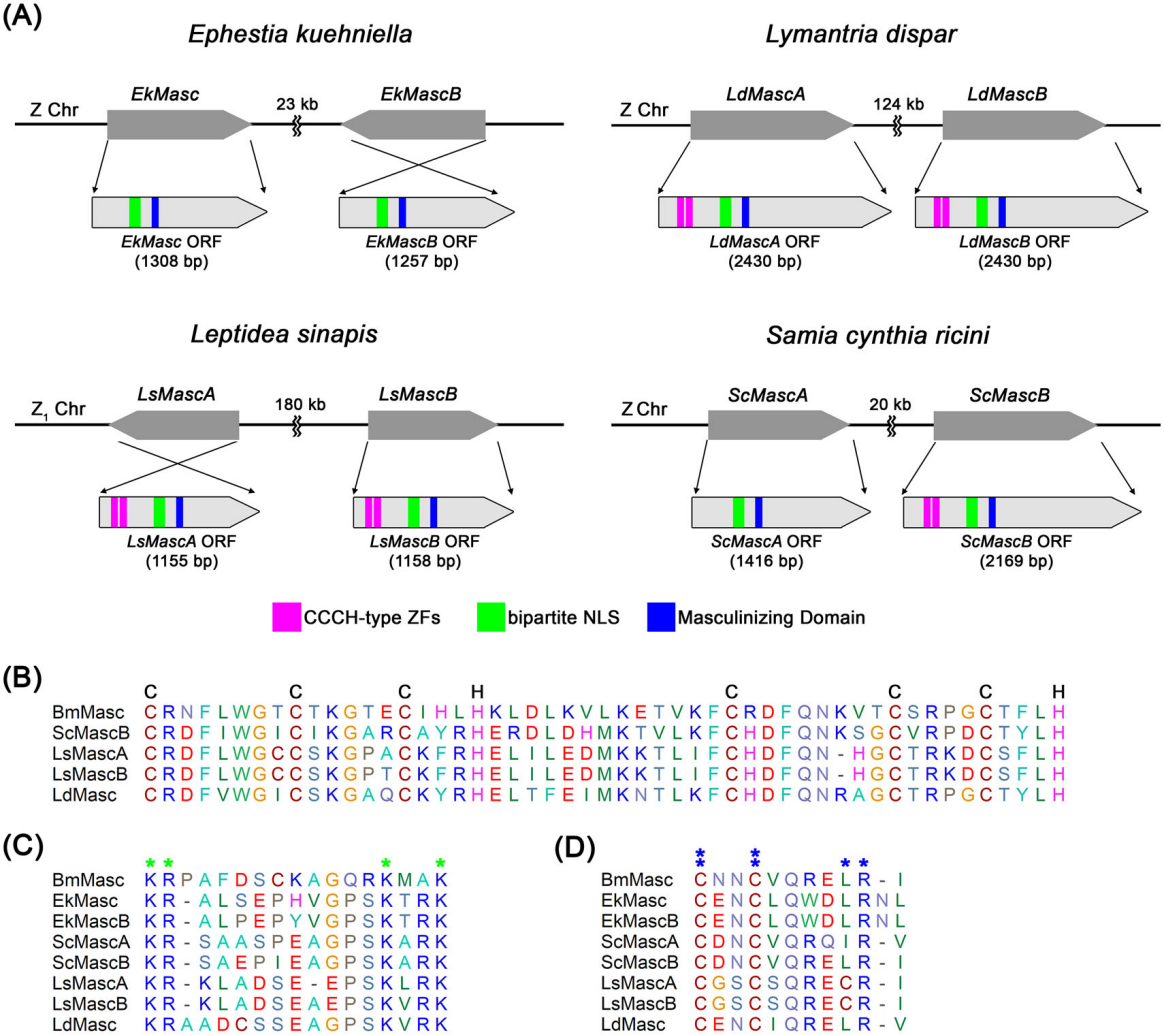
Genomic organization of duplicated *Masc* genes in *Ephestia kuehniella* (*EkMasc* and *EkMascB*), *Lymantria dispar* (*LdMascA* and *LdMascB*), *Leptidea sinapis* (*LsMascA* and *LsMascB*) and *Samia cynthia ricini* (*ScMascA* and *ScMascB*). (A) Schematic representation of the orientation and distance of the duplicated *Masc* genes on the Z chromosome (Z Chr) and the open reading frame (ORF) of each *Masc* transcript indicating the zinc finger domains (CCCH‐type ZFs), the bipartite nuclear localization signal (NLS) domain and the masculinizing domain. (B–D) Alignments of protein sequences of the Masc domains, including the *Bombyx mori* Masc (BmMasc) domains for comparison. LdMasc refers to the LdMascA and LdMascB domains of *L. dispar*, which are identical. (B) CCCH‐type ZFs with the indicated CCCH amino acid residues (not shown for EkMasc, EkMascB and ScMascA because they are missing). (C) Bipartite NLS domain; green asterisks indicate four conserved positively charged residues, lysine (K) and arginine (R), which are important for the function of this domain. (D) Masculinizing domain; double blue asterisks indicate conserved cysteine (letter C) residues, which are essential for masculinization, and single blue asterisks indicate leucine (L) and arginine (R) residues, which influence the efficiency of masculinization in *B. mori*.

The Z‐linkage is one of the important and characteristic features of the *Masc* gene of *B. mori* and the functional orthologs of *Masc* in the species studied. In several species, this gene was found on the Z chromosome near the genes for 6‐phosphogluconate dehydrogenase (6‐PGD) and sterol regulatory element‐binding protein (SREBP) (Visser *et al.*, 2021; Pospíšilová *et al.*, 2023; Van′t Hof *et al.*, 2024) and based on the shared synteny and collinearity of genes between Lepidoptera (Wright *et al.*, 2024), we can expect a similar location of *Masc* genes in other moths and butterflies. The structure of Masc proteins also has characteristic features in the studied species (Fig. 2), namely two zinc finger motifs (but absent in *Ephestia kuehniella*), the bNLS domain and the masculinizing domain (Deng *et al.*, 2021; Visser *et al.*, 2021; Bi *et al.*, 2022; Pospíšilová *et al.*, 2023; Li *et al.*, 2024; Van′t Hof *et al.*, 2024). In addition, the Masc proteins have a relatively high proline content, which is 2–3 times higher compared to other proteins of the studied species. The proline residues are concentrated in two proline‐rich domains, PRD‐1 and PRD‐2. In particular, PRD‐2 at the C‐terminus could be important for the function of Masc, either by ensuring the stability of the protein or by providing binding sites for transcription factors (Visser *et al.*, 2021).

An interesting feature of the *Masc* gene is the alternative splicing. In *B. mori*, the splice variant *Masc‐S* lacks the exon containing the *Fem* piRNA target sequence and is therefore not sensitive to downregulation by *Fem* piRNA. Although it is expressed in both sexes, the results of silencing of *Masc‐S* by RNA interference (RNAi) suggest an important role in the development of the external female genitalia (Zhao *et al.*, 2019). In contrast, male‐specific splice variants that skip the exon essential for masculinization, called *Masc^ms^*, were identified in two pyralid moths, *E. kuehniella* and *Plodia interpunctella*, and in the codling moth *C. pomonella*, where they were found in male somatic tissues but not in the testes (Visser *et al.*, 2021). In the follow‐up study in *C. pomonella*, an exceptional splicing variability of *CpMasc* was found. Of the 14 *CpMasc* splice variants identified, three were male‐biased: one full‐length transcript and two transcripts lacking the exon essential for masculinization (Pospíšilová *et al.*, 2023). A male‐specific splice variant that skips this exon was also identified in *LdMasc* of *Lymantria dispar* (Moronuki *et al.*, 2025). All these results suggest that *Masc* genes in different species may have other functions besides masculinization and dosage compensation.

### Duplicated Masc on the Z chromosome

The real curiosity is the presence of two functional copies of the *Masc* gene in *E. kuehniella*, *EkMasc* and *EkMascB* (Visser *et al.*, 2021). Both the *EkMasc* gene and its paralog *EkMascB*, resulting from a recent duplication, are located on the Z chromosome in opposite orientation (tail‐to‐tail) with a distance of about 23 kb between them and with an ortholog of the *6‐phosphogluconate dehydrogenase* (*Ek6‐Pgd*) gene in close proximity to *EkMasc*. Both genes have a similar structure (Fig. 2A). They contain 12 exons and a similar open reading frame and encode similar proteins with a high sequence identity of 91.7% over the entire length. The EkMasc and EkMascB proteins have lost the double zinc finger domain, which is conserved in other lepidopterans (e.g., Fig. 2B; Kiuchi *et al.*, 2014; Deng *et al.*, 2021; Pospíšilová *et al.*, 2023; Li *et al.*, 2024; Van′t Hof *et al.*, 2024), but they do contain the other two conserved domains, the bNLS domain (Fig. 2C) and the masculinizing domain (Fig. 2D), which show almost complete homology between the two proteins (Visser *et al.*, 2021). Both *EkMasc* and *EkMascB* are expressed during early embryogenesis and both genes follow the same expression pattern depending on the sex of the embryos, that is, their expression starts to increase in male embryos at the beginning of sex determination and peaks a few hours later when the transition from default female‐specific splicing of *Ekdsx* to male‐specific splicing is reached, while expression decreases in female embryos at the same time (Visser *et al.*, 2021). Simultaneous knockdown of *EkMasc* and *EkMascB* using embryonic RNAi resulted in female‐specific splicing of *Ekdsx* in genetic males and a strongly female‐biased sex ratio, probably due to the male‐killing effect caused by failure of dosage compensation (cf. Kiuchi *et al.*, 2014; Tomihara *et al.*, 2022). However, no signs of feminization were observed in the few surviving males (Visser *et al.*, 2021), probably because the knockdown effect of embryonic RNAi is transient and lasts 60 to 80 h after injection, as in the silkworm (Yamaguchi *et al.*, 2011). In summary, all results obtained so far indicate a conserved function of both *EkMasc* and *EkMascB* genes in sex determination. However, the adaptive significance of this duplication remains unclear. We can only speculate that the two *EkMasc* copies may reinforce the key role of this gene in male development and dosage compensation.

Surprisingly, two functional copies of the *LdMasc* gene, *LdMasc‐A* and *LdMasc‐B*, were also identified in the spongy moth *L. dispar japonica* (Moronuki *et al.*, 2025). Both copies are located about 124 kb apart on the Z chromosome, but their open reading frames are aligned in the same direction (Fig. 2A), in contrast to the duplicated *EkMasc* in *E. kuehniella* (Visser *et al.*, 2021). The genomic sequences of the *LdMasc‐A* and *LdMasc‐B* genes are very similar and encode nearly identical LdMasc‐A and LdMasc‐B proteins (Fig. 2B–D), making it virtually impossible to detect differences in their expression and function, if any (Moronuki *et al.*, 2025).

Recently, we identified a similar duplication of a *Masc* ortholog in the wood‐white butterfly *Leptidea sinapis*, a species with a complex system of multiple sex chromosomes W_1–3_Z_1–3_/Z_1–3_Z_1–3_ (female/male) that arose through repeated rearrangements between the sex chromosomes and autosomes, including translocations, fusions and fissions (Yoshido *et al.*, 2020). The two copies of the *LsMasc* gene, *LsMascA* and *LsMascB*, are located on the Z_1_ chromosome, which consists of the *Leptidea* orthologs of most of the Z‐linked genes of *B. mori* and part of the genes of *B. mori* chromosome 17 (Yoshido *et al.*, 2020). Both copies are oriented in opposite directions (head‐to‐head) and are about 180 kb apart (Fig. 2A). The structure of the deduced LsMascA and LsMascB proteins contains all three domains (zinc finger domain, bNLS domain and masculinizing domain; Fig. 2B–D), which are characteristic features of the Masc protein of most lepidopteran species studied. Splice variants were also identified in both *LsMasc* genes, and one of the splice variants is expressed only in some tissues of males. However, the role of *LsMasc* genes and their splice variants in sex determination remains to be investigated.

Using the assembled genome sequence (Lee *et al.*, 2021) and embryonic transcriptomes (Yoshido & Marec, 2023), we also identified duplicated orthologs of the *Masc* gene in the Z chromosome of the eri silkmoth *Samia cynthia ricini*, *ScMascA* and *ScMascB*. As in *L. dispar japonica*, both orthologs are oriented in the same direction (Fig. 2A), but the ScMascA protein, unlike the ScMascB protein, lacks the double zinc finger domain (Fig. 2B). The other two domains are conserved and show high sequence similarity (Fig. 2C, D). However, it should be emphasized that the masculinizing role of the *ScMasc* genes has not yet been proven, although they are the main candidates for promoting male sex determination in *S. c. ricini* (Yoshido & Marec, 2023). Differences in the mutual orientation of duplicated *Masc* genes in different species suggest that the copies arose through different duplication mechanisms.

## Search for an upstream feminizing factor in dominant W systems

It was expected that it would be very difficult to find a feminizing gene like *Fem* in *B. mori* on the W chromosome of other Lepidoptera species, because primary sex‐determining genes in insects have a rapid turnover and are therefore often species‐specific or restricted to a small group of related species (Saccone, 2022). The W chromosome of Lepidoptera is largely composed of heterochromatin (Traut *et al.*, 2007) and tends to accumulate various repetitive DNA sequences, especially retrotransposons (Abe *et al.*, 2005; Traut *et al.*, 2013; Lewis *et al.*, 2021; Hejníčková *et al.*, 2023). Due to the repetitive nature of W chromosomes, male genomes were preferentially sequenced to avoid assembly problems until recently when new next‐generation sequencing (NGS) technologies became available, enabling high‐quality genome assemblies including repetitive DNA sequences (e.g., Lewis *et al.*, 2021; Lee *et al.*, 2024). However, there is still only fragmentary information on the actual composition of the W chromosomes of Lepidoptera. Moreover, homology‐based approaches to identify similar sequences between species are challenging due to accelerated molecular divergence of W chromosomes in the absence of meiotic recombination in Lepidoptera females, leading to rapid loss of homology even between closely related species (Vítková *et al.*, 2007; Yoshido *et al.*, 2013; Zrzavá *et al.*, 2018).

Indeed, the initial results in *T. varians*, a species closely related to *B. mori*, suggested that the primary signal gene differs in these two species, as the *Fem* piRNA binding site was not found in a *Masc* ortholog of *T. varians*, *TvMasc* (Lee *et al.*, 2015). The absence of putative *Fem* orthologs was later confirmed in the assembled W chromosome sequence of *T. varians* (GenBank Acc. No. AP028282) and by deep sequencing of embryonic piRNA libraries of *T. varians*, where no piRNA reads complementary to the *TvMasc* sequence were identified, suggesting a *Fem* piRNA‐independent system of sex determination (Lee *et al.*, 2024). Recently, deep sequencing analysis did not identify any female‐specific small RNAs that would map to *OfMasc* mRNA in *O. furnacalis* from the distant family Crambidae, also demonstrating that the sex determination system is independent of piRNA‐mediated cleavage of *Masc* mRNA; consistent with this finding, *OfMasc* mRNA was not downregulated in female embryos compared to male embryos (Fukui *et al.*, 2023). These results suggested that the *Fem* piRNA/*Masc* pathway of sex determination found in *B. mori* is not fully conserved in other Lepidoptera.

However, a feminizing factor similar to but not orthologous to *Fem* in *B. mori* has been identified in *P. xylostella*, a species at the base of Ditrysia. In this species, a W‐linked locus called *Pxyfem*, which consists of “retrocopies” of the *PxyMasc* gene integrated via long terminal repeat (LTR) retrotransposons, generates a variety of small silencing RNAs (ssRNAs), mainly piRNAs. Some of these piRNAs are complementary to *PxyMasc* mRNA and thus capable of silencing this male‐determining gene by RNAi via a ping‐pong cycle between piRNAs of *PxyFem* and *PxyMasc* (Harvey‐Samuel *et al.*, 2022). Recently, Moronuki *et al.* (2025) used transcriptome analyzes to search for a feminizing factor in *L. dispar japonica* and identified a gene on the W chromosome that meets the criteria for the upstream primary trigger of sex determination. They named this gene *Fet‐W* (*Female expressed transcripts of the W chromosome*). The *Fet‐W* gene consists of about 100 tandemly repeated sequence copies that are aligned in the same direction within a 200 kb region on the W chromosome. The gene is expressed in female embryos, with a peak of expression in the middle of the sex determination period. More importantly, *Fet‐W* has a piRNA cluster‐like structure, similar to *Fem* in *B. mori*, and its RNA transcripts show 100% complementarity to a 21‐nucleotide stretch of *LdMasc* mRNA, suggesting that *Fet‐W* is a piRNA precursor targeting *LdMasc* mRNA. Thus, like *Fem* in *B. mori* (Kiuchi *et al.*, 2014), *Fet‐W* could promote female development by cleaving *LdMasc* mRNA through piRNA biogenesis in female embryos. This conclusion was supported by knockdown of *Fet‐W* expression using embryonic RNAi, which led to an increase in *LdMasc* expression in female embryos and a shift in *Lddsx* splicing from the female to the male variant (Moronuki *et al.*, 2025).

**Fig. 3:**
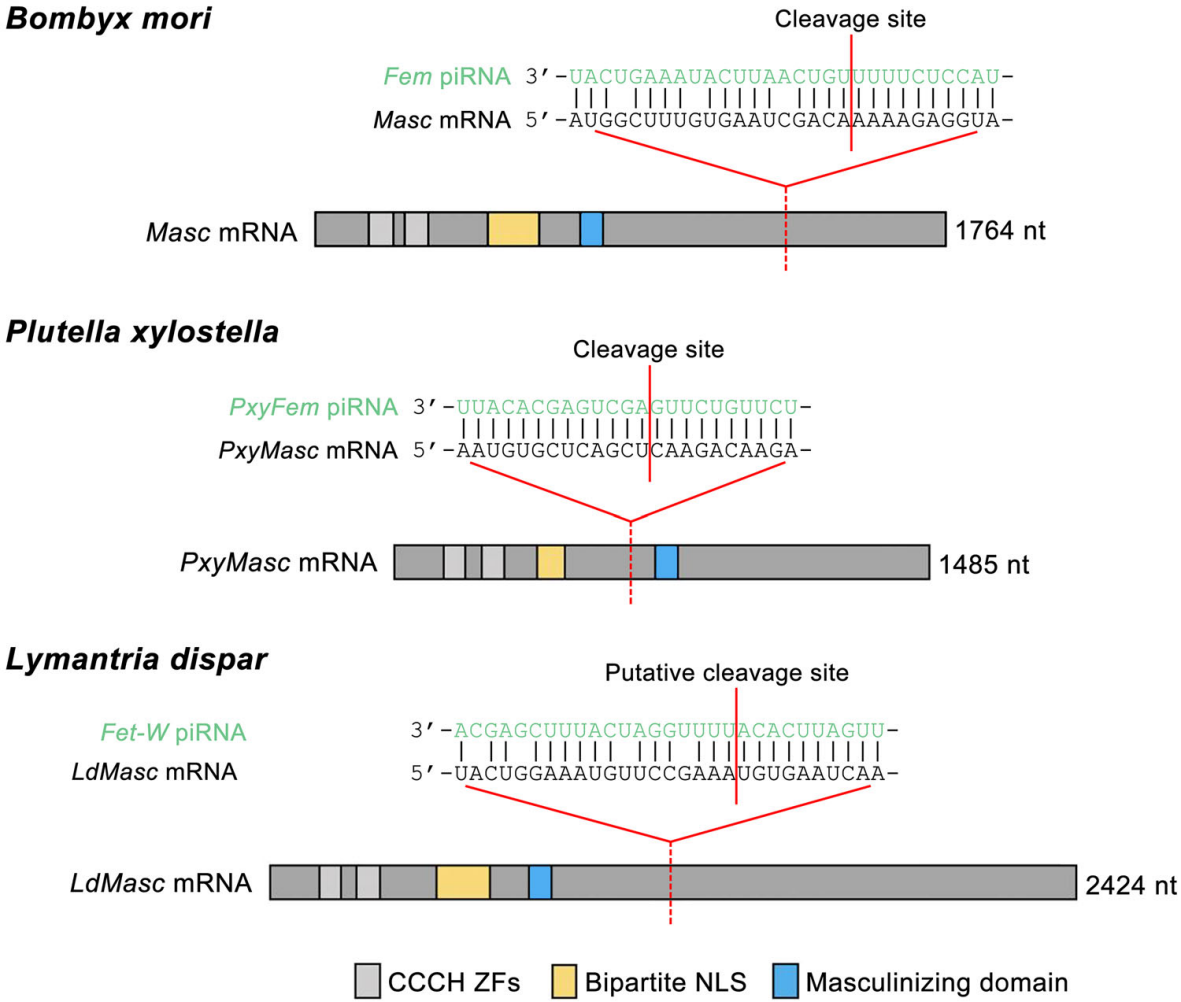
Comparison of primary sex‐determining signals between distantly related Lepidoptera species. Shown are the feminizing piRNAs and their target *Masc* mRNA sequences as well as the position of the cleavage site on the *Masc* gene in *Bombyx mori*, *Plutella xylostella*, and *Lymantria dispar*. [Reproduced with a slight modification from Moronuki *et al.* (2025) and with permission from Masataka G. Suzuki]

The above discoveries show that female sex is controlled by a similar mechanism via piRNA‐mediated RNAi in the silkworm *B. mori*, the diamondback moth *P. xylostella* and the spongy moth *L. dispar* (Kiuchi *et al.*, 2014; Harvey‐Samuel *et al.*, 2022; Moronuki *et al.*, 2025). However, the complete lack of sequence homology between the *Fem*, *PxyFem* and *Fet‐W* genes and between the piRNA‐targeted *Masc* sequences suggests convergent evolution of a similar primary trigger of sex determination in these three phylogenetically distant Lepidoptera species (Fig. 3). An interesting question is the origin of these similar sex determination mechanisms. This question was recently addressed by Lee *et al.* (2024), who sequenced and assembled the W chromosomes of two bombycid moths, *B. mori* with *Fem* and *T. varians* with a *Fem*‐independent system, to decipher the origin of *Fem* in *B. mori*. They found remnants of homology between the Z and W chromosomes, in contrast to the study by Han *et al.* (2024), suggesting that the W chromosome of both species evolved from the Z chromosomes. By analyzing the high‐quality assembly of the W chromosome of *B. mori*, Lee *et al.* (2024) found that a large part of the transcription unit of *Fem* has a high similarity to the RTE‐type LINE BovB transposon, and the first half of the inner *Fem* piRNA‐producing part has a satellite DNA‐like sequence, while the second half has a similarity to the LTR of the gypsy transposon. Based on this finding, the authors proposed that *Fem* is a chimeric sequence formed by the random fusion of multiple transposons whose boundaries happen to form the sequence complementary to *Masc*, which produces *Fem* piRNA. The *Fem* sequence then replicated by mechanisms other than retrotransposition, as all 129 *Fem* copies found in the assembled W chromosome have the same orientation.

Based on current knowledge and the convergent evolution of feminizing factors in three distant species, three hypotheses for their independent origin can be proposed. (1) Assuming that W and Z were originally homologous chromosomes, the feminizer could be an allele of *Masc* that evolved into the piRNA‐producing gene during the genetic erosion of the nonrecombining W chromosome. This hypothesis does not seem likely in *B. mori* (Lee *et al.*, 2024). (2) A feminizer could arise from the transposition of a *Masc* copy to the W chromosome, as shown in *P. xylostella*, where *PxyFem* arose from *PxyMasc* “retrocopies” integrated via LTR retrotransposons (Harvey‐Samuel *et al.*, 2022). (3) A feminizer could arise randomly as a chimeric sequence of multiple transposons, as proposed for *B. mori Fem* (Lee *et al.*, 2024).

## W chromosome‐independent sex determination mechanisms

In Lepidoptera, most species of basal nonditrysian taxa and some species of the clade Ditrysia have a Z0/ZZ sex chromosome system (Marec *et al.*, 2010; Sahara *et al.*, 2012). In these species, a different mechanism for sex determination must have evolved that is independent of the W chromosome. In addition, some reports have shown that even in some species with WZ/ZZ sex chromosome system, females have lost the W chromosome or males have a W chromosome, clearly indicating that the W chromosomes are dispensable for sex determination (Yoshido *et al.*, 2016; Kageyama *et al.*, 2017; Van′t Hof *et al.*, 2024). Therefore, sex determination mechanisms that do not rely on the W chromosome should be more widespread in Lepidoptera. In this section, we briefly present the current state of knowledge on these non‐W chromosome‐based sex determination mechanisms.

### Masc as a primary switch of sex determination in a butterfly

**Fig. 4:**
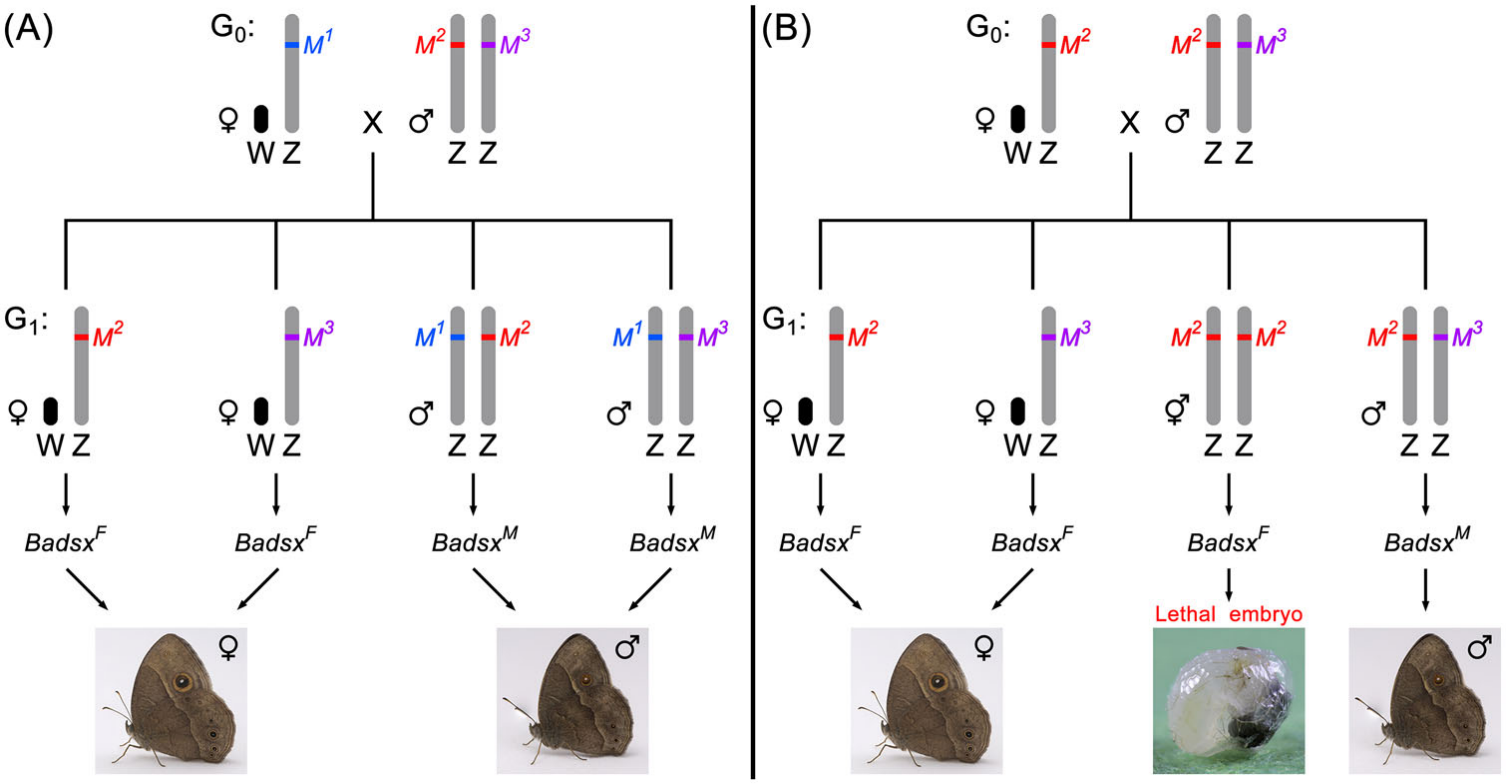
Schematic representation of two crosses between *Bicyclus anynana* females (WZ) and males (ZZ) with different *BaMasc* alleles (*M^1^*, *M^2^* and *M^3^*) on the Z chromosome. G_0_ and G_1_ denote the parental generation and the generation of the offspring, respectively. *Badsx^F^* and *Badsx^M^* are the female and male splice variants of the *B. anynana doublesex* gene. (A) A cross with three different alleles results in fully viable offspring: females are hemizygous and males are heterozygous for *BaMasc*. (B) A cross with two different alleles results in viable female offspring, but half of the male offspring are homozygous for *BaMasc* (*M^2^M^2^*) and die in late embryogenesis.

Compared to the moths, the butterflies (superfamily Papilionoidea) have been somewhat neglected in studies on sex determination. When studying sex‐linked embryonic lethality in the butterfly *Bicyclus anynana* (Nymphalidae: Satyrinae), a model for evolutionary and ecological genetics, Van′t Hof *et al.* (2024) made a surprising discovery. They found that sexual development in this species is controlled by a fundamentally different mechanism than the *Fem* piRNA/*Masc* pathway in *B. mori* (Kiuchi *et al.*, 2014). This mechanism is based on the number of alleles or allelic heterozygosity of the Z‐linked *B. anynana Masc* (*BaMasc*) gene. WZ embryos with a single Z chromosome and therefore with a single *BaMasc* allele (i.e., hemizygotes) produce the female *Badsx* isoform and develop into females, whereas ZZ embryos with two distinct *BaMasc* alleles (i.e., heterozygotes) produce the male *Badsx* isoform and develop into males. However, ZZ embryos with two identical *BaMasc* alleles (i.e., homozygotes) produce the female isoform of *Badsx*, resulting in an intersex condition and failure of dosage compensation, and die in late embryogenesis (Fig. 4). Thus, *BaMasc* itself is the primary sex‐determining switch that does not require any upstream initiating factor. Furthermore, the W chromosome is not required for female development, as demonstrated by the detection of viable and reproductively competent Z0 females (Van′t Hof *et al.*, 2024).

This unique sex‐determining mechanism functions via sequence diversity in exons 8 and 9 of the *BaMasc* gene, which is caused by single nucleotide substitutions (synonymous and nonsynonymous) and indels (insertions or deletions). Exon 8 contains a hypervariable region (HVR) whose protein sequence consists mainly of asparagine (N). The length of the HVR varies greatly and ranges from 7 to 24 amino acids. Exon 9 is less variable in length and its protein sequence ranges from 94 to 102 amino acids. However, this exon exhibits considerable nucleotide diversity, resulting in a high level of amino acid polymorphism. Heterozygosity of exons 8 and 9 in ZZ embryos results in the production of two distinct BaMasc proteins that likely form a heteromeric complex required for male‐specific *Badsx* splicing. Hemizygosity (WZ embryos) or homozygosity (ZZ embryos) of these exons leads to the inactive homomeric BaMasc protein, which by default leads to female‐specific *Badsx* splicing. However, how the embryo distinguishes between the homomeric and heteromeric state of the BaMasc protein is not yet known (Van′t Hof *et al.*, 2024). Theoretically, such a recognition mechanism could be based on binding differences between different and identical protein variants that would activate or inactivate the heteromeric or the homomeric protein complex, as recently demonstrated by the mechanism of sex determination in the honeybee *Apis melifera* (Otte *et al.*, 2023; Seiler & Beye, 2024).

The sex‐determining mechanism recently discovered in *B. anynana* (Van′t Hof *et al.*, 2024) is surprisingly more similar to that of the honeybee (Beye *et al.*, 2003), which belongs to a different insect order, Hymenoptera, than to that of the silkworm (Kiuchi *et al.*, 2014). In the haplodiploid system of *A. mellifera*, sex determination is controlled by the *complementary sex determiner* (*csd*). The Csd and BaMasc proteins have similar HVRs characterized by a similar composition of amino acids with a predominance of asparagine. Diploid heterozygotes for the *csd* locus become viable females and haploid hemizygotes become viable males. However, diploids that are homozygous for *csd* develop into males and are eaten by the workers (Beye *et al.*, 2003). The similarity of these mechanisms in *B. anynana* and *A. mellifera* is a clear example of convergent evolution.

In zygosity‐based sex determination of *B. anynana*, individuals die as embryos if they inherit two identical alleles of *BaMasc* from their parents. However, such a situation is probably very unusual in natural populations, as continuous selection against homozygotes leads to an exceptionally high haplotype diversity of *BaMasc*. Indeed, in a large‐scale population study, 205 different coding sequences of *BaMasc* were found in a sample of 246 females of *B. anynana* (Van′t Hof *et al.*, 2024). Nevertheless, the probability of inheriting two identical alleles leading to embryonic death would be much higher in severely declining populations with reduced genetic variation due to inbreeding. This mechanism of sex determination could therefore have implications for conservation genetics.

### Sex determined by the Z:A ratio

The geographic subspecies of wild silkmoths, *Samia cynthia* ssp. (Saturniidae), exhibit unique variations in the sex chromosome systems, including Z0/ZZ in *S. c. ricini*, neo‐Wneo‐Z/neo‐Zneo‐Z in *S. c. walkeri*, neo‐WZ_1_Z_2_/Z_1_Z_1_Z_2_Z_2_ in *S. c*. subsp. indet., and WZ/ZZ in *S. c. pryeri* (Yoshido *et al.*, 2013). A genetic study clearly showed that males always have two Z (or neo‐Z) chromosomes and females have one Z (or neo‐Z) chromosome, regardless of the presence or absence of the W (or neo‐W) chromosome, suggesting that the W and neo‐W chromosomes are not required for sex determination and reproduction (Yoshido *et al.*, 2016). Subsequent sex determination experiments with polyploids induced by heat and cold shock during early embryogenesis showed that tetraploids with four Z chromosomes become males and tetraploids with two Z chromosomes become females (Yoshido & Marec, 2023). In addition, crosses between diploids and tetraploids produced triploids with three Z chromosomes and triploids with two Z chromosomes. Triploids with three Z chromosomes became males, while triploids with two Z chromosomes exhibited both male‐ and female‐specific splicing of *Scdsx* and had abnormal gonads, suggesting that triploids with two Z chromosomes are intersexes. These results showed that deviations in the ratio between the number of Z chromosomes and the number of autosome sets (Z:A ratio) caused by ploidy changes disrupt sexual development, clearly demonstrating that sex is determined by the Z:A ratio and not only by the Z chromosome number (Table 2). In addition, embryonic transcriptome analyzes showed that the relative levels of gene expression are similar between samples with different doses of Z chromosomes and autosome sets. These results suggest that (i) dosage compensation of Z‐linked genes was not affected and (ii) there is a ploidy‐associated dosage compensation mechanism to compensate for the different doses of Z chromosomes and autosomes caused by ploidy changes (Yoshido & Marec, 2023). The exact molecular mechanism of sex determination in *S. cynthia* remains to be elucidated.

**Table 2:** Sexual development of polyploids and aneuploids in *Bombyx mori* (W‐dominant sex determination)^†^ and *Samia cynthia ricini* (W‐independent sex determination)^‡^

Sex	Z:A ratio	2*n*	3*n*	4*n*
*Bombyx mori*				
Female Female Female Male Female Male	0.33 0.5 0.67 0.67 0.75 1.0	WZ + 27AA ZZ + 27AA	WWZ + 27AAA WZZ + 27AAA, WWZZ + 27AAA ZZ + 27AAA ZZZ + 27AAA	WWZZ + 27AAAA WZZZ + 27AAAA ZZZZ + 27AAAA
*Samia cynthia ricini*				
Female Intersex Male	0.5 0.67 1.0	Z0 + 13AA ZZ + 13AA	ZZ0 + 13AAA ZZZ + 13AAA	ZZ00 + 13AAAA ZZZZ + 13AAAA

^†^ Data from Kawamura (1988), Sahara *et al.* (1997), and Tazima (1964).

^‡^ Data from Yoshido & Marec (2023).

## Concluding remarks and research perspectives

**Fig. 5:**
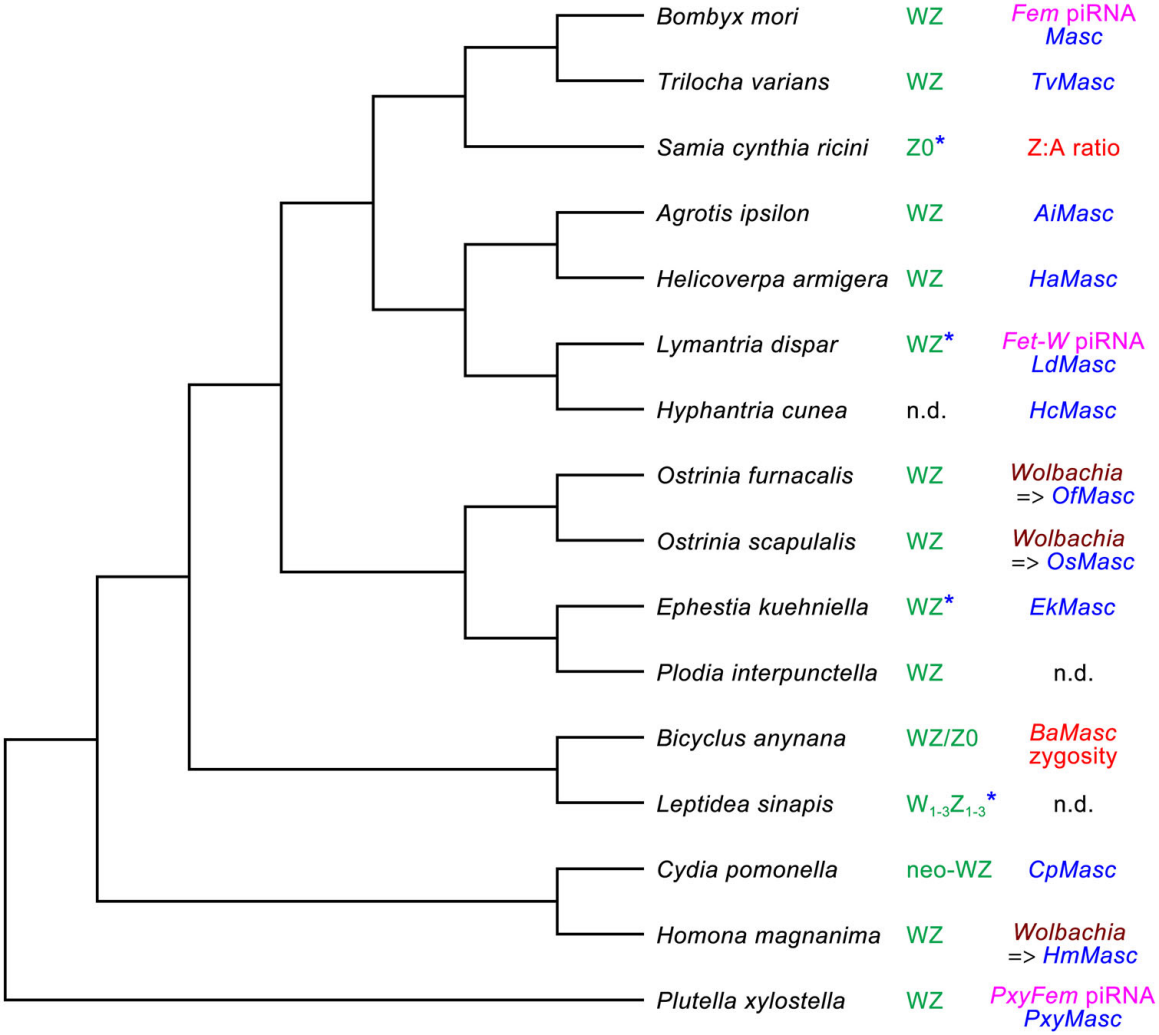
A simple tree showing the phylogenetic relationships between all Lepidoptera species discussed in this review with respect to sex determination. The cladogram is based on a recent comprehensive phylogeny of the Lepidoptera (Kawahara *et al.*, 2019) and shows for each species the currently available information on the constitution of sex chromosomes in females (green) and the mechanism of sex determination. Feminizing piRNAs that control female development are shown in pink, *Masc* genes required for male development in blue and *Wolbachia* downregulating *Masc* genes in brown. Fundamentally different mechanisms of sex determination based on the Z:A ratio or zygosity of the *BaMasc* gene are shown in red. Blue asterisks at the sex chromosome symbols indicate species with a duplicated *Masc* gene, n.d. means not determined.

Moths and butterflies (Lepidoptera) are a very diverse and species‐rich group of insects, comprising almost 160 000 described species (Van Nieukerken *et al.*, 2011). It is therefore highly speculative to generalize knowledge about the mechanisms of sex determination when only a few species have been studied in this respect. Nevertheless, the available data suggest that the key role of the *Masculinizer* gene in male sex determination is conserved at least in the largest clade Ditrysia with >150 000 species (Kawahara *et al.*, 2019). The masculinizing function of *Masc* has been clearly demonstrated in species across the phylogenetic tree (Fig. 5), including *P. xylostella* and *C. pomonella* from the basal groups of Ditrysia, the Yponomeutoidea and Tortricoidea (Harvey‐Samuel *et al.*, 2020; Pospíšilová *et al.*, 2023). Induction of dosage compensation by the Masc protein has been demonstrated in *B. mori* and *O. furnacalis* (Katsuma *et al.*, 2022; Tomihara *et al.*, 2022), and embryonic lethality upon suppression or malfunction of *Masc* has been attributed to failure of dosage compensation in evolutionarily distant Lepidoptera species (e.g., Harvey‐Samuel *et al.*, 2020; Van′t Hof *et al.*, 2024). Besides *Masc*, the regulation of sexual differentiation by sex‐specific splicing of the *doublesex* gene is undoubtedly conserved in Lepidoptera as well as in many other insects (Geuverink & Beukeboom, 2014; Saccone, 2022). In contrast, the limited data obtained to date suggest that in species with the presumably dominant W chromosome, the primary sex‐determining signals (i.e., feminizing factors) are not conserved, consistent with the large diversity of upstream signals/genes in insect sex‐determining pathways (Saccone, 2022). However, a similar mechanism of female sex determination has convergently evolved in three very distantly related species (*B. mori*, *L. dispar*, and *P. xylostella*; Fig. 5). It is based on feminizing piRNAs that silence the expression of *Masc* genes, although they are produced by different W‐linked genes (Kiuchi *et al.*, 2014; Harvey‐Samuel *et al.*, 2022; Moronuki *et al.*, 2025). On the other hand, the apparent absence of feminizing piRNAs in several other species such as *T. varians* and *O. furnacalis* (Fukui *et al.*, 2023; Lee *et al.*, 2024) suggests that the evolution of sex‐determining pathways occurs through the recruitment of different primary triggers of sex determination, which remain to be determined.

**Fig. 6:**
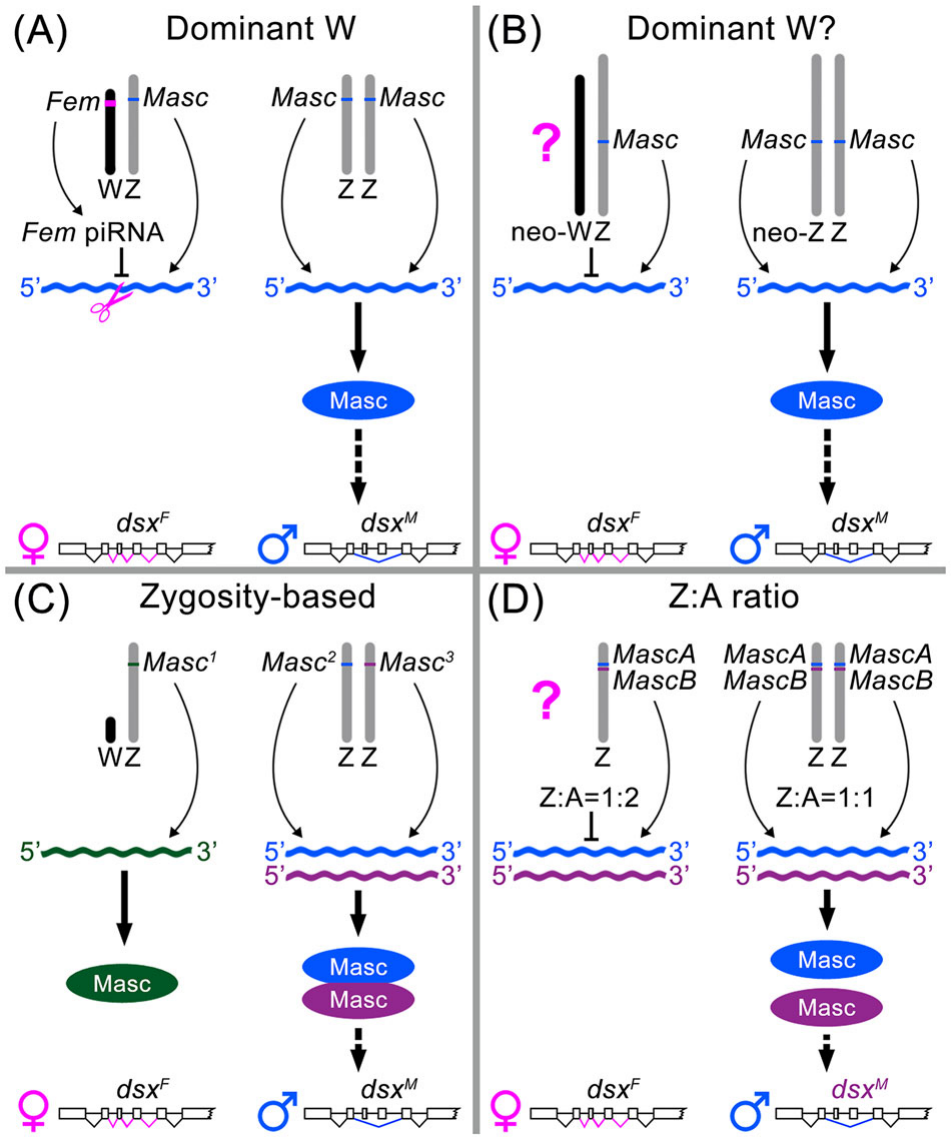
Mechanisms of sex determination in the Lepidoptera. (A) A *Fem* piRNA/Masc pathway discovered in *B. mori* with a dominant W chromosome in WZ females carrying a *Feminizer* (*Fem*) gene produces *Fem* piRNA that downregulates the expression of a Z‐linked gene, *Masculinizer* (*Masc*), leading to female‐specific splicing of the *doublesex* gene (*dsx^F^*) and female development. In ZZ males, the *Masc* gene is fully expressed and the Masc protein promotes male‐specific splicing of *dsx* (*dsx^M^*), leading to male development (Kiuchi *et al.*, 2014). (B) A similar mechanism with the presumably dominant W chromosome, in which the expression of *Masc* is controlled by a yet unknown factor, as found for example in *Cydia pomonella* with neo‐sex chromosomes (Pospíšilová *et al.*, 2023). (C) A zygosity‐based mechanism discovered in the butterfly *B. anynana* depends on the allelic combinations of the Z‐linked *Masc* gene. One copy of *Masc* in hemizygous embryos (WZ or Z0) results in a monomeric Masc protein, the *dsx^F^* splice variant and female development, while two different *Masc* copies in ZZ embryos result in a heteromeric Masc protein that promotes the *dsx^M^* splice variant and male development (Van′t Hof *et al.*, 2024). (D) The mechanism of the Z:A ratio, as demonstrated in *Samia cynthia ricini* without a W chromosome but with a duplicated Masc, depends on the number of Z chromosomes relative to the number of autosome sets. The ratio of 1:2 leads to *dsx^F^* splicing and female development, while the ratio of 1:1 leads to *dsx^M^* splicing and male development (Yoshido & Marec, 2023).

To summarize the previous findings, there are at least three fundamentally different mechanisms of sex determination in the Lepidoptera. (i) In species with a dominant W chromosome, a primary signal gene is actually a female‐determining gene located on the W chromosome; this gene controls the expression of the Z‐linked *Masc* gene either via generated piRNA (Fig. 6A) or by other means (see above; Fig. 6B). (ii) In species without a W chromosome or with a dispensable W chromosome such as *S. c. ricini*, sex is determined by the Z: A ratio (Fig. 6D), but a molecular mechanism for counting the number of Z chromosomes and the number of autosome sets is not yet known (Yoshido & Marec, 2023). (iii) Zygosity‐based sex determination was discovered in the butterfly *B. anynana*, where the primary signal is the state of the hypervariable *Masc* gene (Fig. 6C; Van′t Hof *et al.*, 2024).

Although great progress has been made in the last 10 years in understanding the molecular mechanisms of sex determination in the Lepidoptera, many questions remain unanswered. For example, except in the silkworm, nothing is known about genes that mediate the regulation of male‐specific splicing of *dsx* by *Masc*, such as the Z‐linked *BmImp* gene, whose sex‐specific expression is controlled by its autoregulatory function and thus serves as a memory device for male sex determination (Suzuki *et al.*, 2014), or the autosomal *BmPsi* gene, which is expressed in both sexes but is a key auxiliary factor in male sex determination (Xu *et al.*, 2017). Since the duplication of the *Masc* gene appears to be relatively common, as it has already been detected in four evolutionarily distant species (Visser *et al.*, 2021; Moronuki *et al.*, 2025; Fig. 2), it would be useful to learn more about the mechanism of this duplication and its adaptive significance for male sex determination. In addition, the identification of primary signal genes in more species from different branches of the phylogenetic tree would help to better understand the evolution of sex determination in Lepidoptera. We also know nothing about the exact molecular mechanism of sex determination in species where sex is determined by the Z:A ratio. Furthermore, the origin of zygosity‐based sex determination discovered in the butterfly *B. anynana*, where *BaMasc* acts as a primary switch, remains to be elucidated, as well as the molecular recognition mechanism to distinguish between hemizygous, heterozygous, and homozygous states of *BaMasc*.

The caterpillars of many lepidopteran species are considered important pests in agriculture, forestry and stored products. Therefore, there is a compelling reason to investigate the genetic basis of sex determination, particularly in economically important pest species, as genes controlling sexual development or sexual differentiation can potentially be used to develop genetic pest control methods, as recently discussed in a comprehensive review by Ashmore *et al.* (2025). Detailed knowledge of the sex‐determining pathway could also facilitate the development of genetic sexing strains (GSS) to improve the applicability of the sterile insect technique (SIT) and the derived technique based on inherited sterility (IS). Current SIT/IS programs for the control of lepidopteran pests are based on bisexual releases. However, the use of GSS in certain species would allow only males to be released, which would bring significant economic benefits by reducing operational costs and increasing the efficiency of sterile males (Marec *et al.*, 2005; Marec & Vreysen, 2019).

Genetic technologies targeting the genes of the sex‐determining pathway to develop genetic control methods or GSS in lepidopteran pests are available, such as transgenesis and gene editing. Transgenesis using a *piggyBac* transposable element is well established and successful germline transformation has been demonstrated in the silkworm as well as in several pest species (e.g., Peloquin *et al.*, 2000; Tamura *et al.*, 2000; Martins *et al.*, 2012; Ma *et al.*, 2013). In terms of gene editing, the CRISPR/Cas9 (clustered regularly interspaced short palindromic repeats/CRISPR‐associated protein 9) technique has already been successfully established in several species and used to study gene function (e.g., Kiuchi *et al.*, 2019; Wang *et al.*, 2019a; Wang *et al.*, 2019b; Wei *et al.*, 2025). However, the question arises as to which known sex‐determining genes are suitable for genetic pest control aimed at population suppression or genetic sexing with the aim of eliminating females. Although conditional silencing of the W‐linked gene that produces the feminizing piRNA would lead to lethality of female embryos, it is only applicable in laboratory experiments as it is difficult to achieve due to the repetitive nature of this gene (Kiuchi *et al.*, 2014; Moronuki *et al.*, 2025). The *Masc* gene also does not appear to be a suitable target, as its knockout would lead to death in male embryos, but not in female embryos. However, *Masc* mutations in the target sequence for the feminizing piRNA would result in insensitivity to downregulation of *Masc* and likely lead to female lethality due to dysregulation of dosage compensation. Such a piRNA‐resistant *Masc* gene (*Masc‐R*) was constructed in the silkworm, and transgenic strains expressing *Masc‐R* indeed resulted in lethality of female larvae and partial sex reversal from female to male (Katsuma *et al.*, 2015; Sakai *et al.*, 2016). Nevertheless, the *dsx* gene, whose function and sex‐specific splicing is likely conserved in all Lepidoptera, currently appears to be the best target for genetic pest control as well as for the development of GSS for SIT/IS applications against lepidopteran pests. The *dsx* ortholog of the pink bollworm *Pectinophora gossypiella*, *Pgdsx*, has already been successfully used to construct transgenic sexing strains in three species, *B. mori*, *P. xylostella* and *P. gossypiella* (Jin *et al.*, 2013; Tan *et al.*, 2013). In these strains, conditional female lethality is driven by the tetracycline‐repressible transactivator (tTAV) protein, which is only expressed in females due to the female‐specific spliced region of *Pgdsx*. However, we hope that progress in understanding the sex‐determining pathway in Lepidoptera will soon reveal other relevant genes and new opportunities for the controlled manipulation of sexual development in species of interest.

## Disclosure

The authors have no conflicts of interest to declare.
